# Antiviral Activity of the Marine Haptophyta *Diacronema lutheri*

**DOI:** 10.3390/md23010012

**Published:** 2024-12-28

**Authors:** Eleonora Montuori, Annalisa Ambrosino, Gerardo Della Sala, Costanza Ragozzino, Gianluigi Franci, Carla Zannella, Anna De Filippis, Donatella de Pascale, Massimiliano Galdiero, Chiara Lauritano

**Affiliations:** 1 Department of Chemical, Biological, Pharmaceutical and Environmental Sciences, University of Messina, Viale F. Stagno d’Alcontres 31, 98166 Messina, Italy; eleonora.montuori@studenti.unime.it (E.M.); costanza.ragozzino@studenti.unime.it (C.R.); 2 Ecosustainable Marine Biotechnology, Stazione Zoologica Anton Dohrn, Via Acton 55, 80133 Napoli, Italy; gerardo.dellasala@szn.it (G.D.S.); donatella.depascale@szn.it (D.d.P.); 3 Department of Experimental Medicine, University of Campania “L.Vanvitelli”, Via De Crecchio, 7, 80138 Naples, Italy; annalisa.ambrosino@unicampania.it (A.A.); carla.zannella@unicampania.it (C.Z.); anna.defilippis@unicampania.it (A.D.F.); massimiliano.galdiero@unicampania.it (M.G.); 4 Department of Medicine, Surgery and Dentistry “Scuola Medica Salernitana”, University of Salerno, 84081 Salerno, Italy; gfranci@unisa.it

**Keywords:** antiviral activity, *Diacronema lutheri*, anticancer, glycerolipids

## Abstract

There are still several viral infections affecting a considerable number of the world’s population, causing thousands of deaths each year. There are no drugs available for most viral infections and for many not even a vaccine. The marine kingdom is characterized by a huge chemical diversity; however, there is currently on the market only one drug derived from the sea with antiviral properties, called Ara-A. In the current study, we used a solid phase extraction method (SPE) to obtain pre-purified fractions from *Diacronema lutheri* raw extracts. We tested both raw extracts and fractions against enveloped and non-enveloped viruses. Results showed an antiviral activity of fraction C of *D. lutheri* against the herpes simplex virus type 1 (HSV-1 strain SC16). Liquid chromatography coupled with untargeted high-resolution tandem mass spectrometry (LC-HRMS^2^) were employed to chart the metabolite distribution in all SPE fractions and pinpoint molecular families unique (or almost unique) to the bioactive fraction. Sulfoquinovosyl di- and monoacylglycerols (SQDGs and SQMGs) and di- and monogalactosyl monoacylglycerols (DGMGs and MGMGs) represent the largest groups of compounds in fraction C and they are likely responsible for the antiviral properties of this fraction.

## 1. Introduction

Despite several investigations with the aim of understanding viral physiology and appropriate treatments and vaccines, there are still several infections affecting a considerable number of the worldwide population, causing thousands of deaths annually. There are no available drugs for most viral infections and not even a vaccine. Additional studies are, hence, required to find successful bioactive molecules as alternative therapies. Even if the marine realm is characterized by a huge chemical diversity, currently on the market, there is only one marine-derived drug with antiviral properties, named Ara-A, while another one is in phase I clinical trials, named Griffithsin [1]. Ara-A, also named Vidarabine, is a nucleoside originally extracted from a sponge, approved by the Food and Drug Administration (FDA) in 1976, and actually used for the treatment of herpes simplex virus (HSV). Griffithsin is a lectin originally isolated from a red alga and shown to have potential anti-HIV activity.

Marine microalgae are considered potentially new sources of biologically active compounds for different human diseases such as cancer, diabetes [2], tuberculosis [3], inflammation [4] and viral infections [5,6]. Regarding antiviral molecules from microalgae, up to now few have been reported. In particular, exopolysaccharides (EPS) from *Porphyridium purpureum* (formerly *Porphyridium cruentum*) (Rhodophyta) which reduced virus-induced cytopathogenicity (herpes simplex virus, vescicular stomatitis virus and vaccinia virus), monogalactosyl diacylglyceride from *Coccomyxa* sp. (Chlorophyta) KJ which induce structural changes in virus particles (HSV), a Marennine-like pigment from *Haslea karadagensis* (Bacillariophyceae) and the polysaccharide-rich fraction of *Dunaliella salina* (Chlorophyta) which are able to inhibit the plaque formation of the HSV, and a sulfated polysaccharide p-KG03 from *Gyrodinium impudium* (Dinoflagellata) which targeted virus particle attachment to cell surface receptors and internalization via virus–cell fusion for influenza A virus H1N1 and H3N2 [7,8,9,10,11].

In the current study, we investigated the antiviral activity of the raw extracts and the derived five fractions of the marine microalga *Diacronema lutheri* (Haptophyta) against two different types of viruses, *herpes simplex virus type 1* (HSV-1 strain SC16) and *poliovirus type 1* (PV-1), respectively, an enveloped and non-enveloped virus. The Herpesviridae family has been reported to include hundreds of different viruses, and nine of them are known to induce disease in humans [12,13]. According to the World Health Organization, 3.7 billion people under age 50 globally have a herpes simplex virus type 1 infection [14]. HSV-1 commonly affects oral mucosa causing herpes labialis or oral herpes, but it can also cause genital mucosa infection resulting in genital herpes [13,15]. The HSV viral particle is structurally composed of an icosahedral capsid consisting of 162 capsomers wrapped in an outer envelope consisting of a double-layered lipid envelope with branched glycoproteins incorporated from the viral genome. The capsid contains a double-strand viral DNA genome [12,13]. Poliovirus belongs to the Picornaviridae family and are non-enveloped RNA viruses. There are three serotypes of poliovirus such as PV1, PV2 and PV3 [16]. Poliovirus 1 causes poliomyelitis that affects children under 5 years of age [15]. The viral particle of poliovirus is morphologically composed of an icosahedral structure determined by the arrangement of proteins in the capsid [17].

The haptophyte, flagellate microalga, *Diacronema lutheri* CCMP1325 (formerly known as *Pavlova lutheri* [18]) has been reported as a key organism for studying microalgal lipid biosynthesis [19] and it is also used in aquaculture [20]. In particular, *D. lutheri* has been shown to be rich in the long-chain polyunsaturated fatty acids n-3 series n-3 LC-PUFAs [21] and in phytosterols [22]. Recently, in 2021, Hulatt et al. also sequenced its nuclear genome with an assembly size of 43.503 Mb and 14,446 protein-coding genes [19].

Regarding the bioactivities of *D. lutheri*, Oh et al. [20] purified a novel peptide (Met-Gly-Arg-Tyr) with inhibitory properties against α melanocyte stimulating hormone (MSH)-induced melanogenesis via melanin content and tyrosinase (TYR) inhibition in mouse B16F10 melanoma cells [20]. Ko et al. [23] isolated from *P. lutheri* (KMCC H-006) a heptameric peptide (Leu-Leu-Ala-Pro-Pro-Arg) able to inhibit the matrix metalloproteinase-9 (MMP-9) protein [23]. High levels of MMPs are strictly related to tumor progression [24]. This heptameric peptide inhibited the MMP-9 at mRNA and protein level in phorbol 12-myristate 13-acetate (PMA)-stimulated HT1080 fibrosarcoma cells with the suppression of phosphorylation of IκB-α, c-Jun N-terminal (JNK) and p38 mitogen-activated protein kinases [23]. *P. lutheri* lipid extracts also showed an anti-inflammatory activity by down-regulating the pro-inflammatory cytokine IL-6 after 24 h of exposure in lipopolysaccharide (LPS)-stimulated THP-1 macrophages [25].

In the current study, after bioactivity testing of raw extracts and fractions against the *herpes simplex* virus type 1 (HSV-1 strain SC16) and *poliovirus* type 1 (PV-1), respectively, an enveloped and non-enveloped virus, the most bioactive fraction was subjected to chemical analysis by using mass spectrometry to identify the class of compounds associated with the observed antiviral activity.

## 2. Results

### 2.1. Experimental Set Up for Diacronema lutheri

The first step of our study was to culture the microalga *Diacronema lutheri* in large quantities (replicates of 10 L cultures) in order to obtain enough biomass for bioactivity screening and chemical extraction. In order to avoid metabolic changes between replicates and allow the production of the same metabolites, culturing conditions were optimized and standardized for *D. lutheri*. In particular, cultures were kept in a climate chamber at 19 °C on a 12:12 h light/dark cycle at 100 μmol photons m^−2^ s^−1^. In addition, we have harvested the microalgal cells always in the same growth phase (i.e., stationary phase).

### 2.2. Citotoxicity on Vero Cells

**Figure 1:**
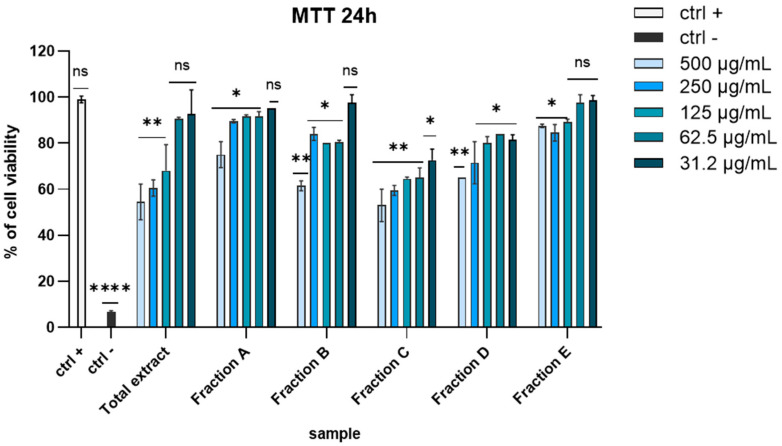
MTT assay on VERO cells after 24 h of exposition. Concentrations are expressed in μg/mL. Positive control (ctrl +): untreated cells. Negative control (ctrl −): dimethyl sulfoxide (DMSO) 100%. **** *p* < 0.0001, ** *p* < 0.0064, * *p* < 0.0221, ns: not statistically significant.

Before exploring the antiviral potential of *D. lutheri* raw total extract and fractions, we evaluated possible cytotoxicity by 3-(4,5-dimethylthiazol-2-yl)-2,5-diphenyl-tetrazolium bromide (MTT) assay on Vero cells, the first cell line approved by the WHO (World Health Organization) for the production of vaccines [26]. Different concentrations of each extract (ranging from 500 to 31.2 μg/mL) were tested after 24 h of exposure. As shown in Figure 1, fixing a threshold line at 50% none of the extracts significantly affected cell viability (Figure 1).

### 2.3. Antiviral Activity

**Figure 2:**
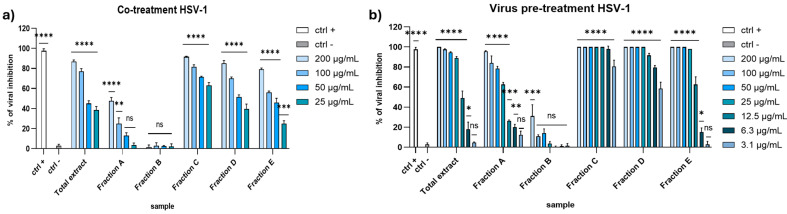
Antiviral activity against HSV-1 in (**a**) co-treatment assay and (**b**) virus pre-treatment. Concentrations are expressed in μg/mL. Positive control (ctrl +): [27]; 100 μg/mL of *Vitis vinifera* leaf extract. Negative control (ctrl −): infected and untreated cells. **** *p* < 0.0001, *** *p* < 0.0006, ** *p* < 0.023, * *p* < 0.0306, ns: not statistically significant.

To explore the antiviral potential of the raw total extract and five fractions, we evaluated the effect against HSV-1 and PV-1 as enveloped and non-enveloped viruses, respectively. As shown in Figure 2a, in the co-treatment assay almost all the fractions showed potent inhibitory activity against HSV-1 at the higher concentrations, except for fractions A and B, which were unable to inhibit the viral replication. In detail, total extract reached 77% inhibition at 100 µg/mL, while the fractions displayed a less powerful activity, except fraction C. Surprisingly, the antiviral effect of fraction C was higher than total extract, recording 81.5% inhibition at 100 µg/mL. In the virus pre-treatment experiment (Figure 2b), we observed a general increase in antiviral activity, maintaining an inhibition greater than 80% at 25 µg/mL of extract. The activity of fraction A was more potent compared to the co-treatment assay, while fraction B was still inactive. Also in this case, fraction C was the most active, exhibiting an IC_90_ equal to 5 µg/mL. These results suggest that the antiviral effect depends on the direct interaction between the compound and virus and occurs before the viral entry.

**Figure 3:**
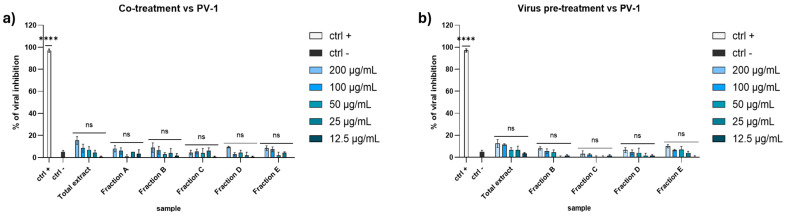
Antiviral activity against PV-1 in (**a**) co-treatment assay and (**b**) virus pre-treatment. Concentrations are expressed in μg/mL. Positive controls (ctrl +): pleconaril 2 μg/mL. Negative control (ctrl −): infected and untreated cells. **** *p* < 0.0001, ns: not statistically significant.

Regarding the activity against the PV-1, none of the extracts seemed to affect the viral replication (Figure 3). These data suggested that the algal extracts could block the enveloped viruses spread and not the non-enveloped ones, probably because they interacted with the envelope during the early stages of infection, thus preventing the virus entry.

The next step was a preliminary exploration of the mechanism of action. To further investigate how the extracts interacted with the viral particles, we performed additional experiments designed to understand in which stage of the initial phases of the infection the extracts could act, either the attachment, or the next stage, the viral entry. The crucial factor that allows us to differentiate between the two phases is the temperature. At 4 °C, indeed, the virus is able to bind to the cell surface but not to enter. At 37 °C, the virus recovers its ability to enter the target cell.

In this regard, we performed the attachment assay to evaluate the extract’s potential to interfere with the binding of the virus to the cell surface. We incubated Vero cells with both virus and extracts at 4 °C to allow the attachment, but not the viral entry. After 1 h, we washed the monolayer and incubated it at 37 °C to allow the viral entry. At the same time, we performed an entry assay to check whether the algal extracts could block the intracellular entry of HSV-1. To do this, we infected the monolayer with the virus and incubated it at 4 °C, allowing the attachment without any type of interference. Then, we treated the monolayer with the extracts and incubated it at 37 °C.

**Figure 4:**
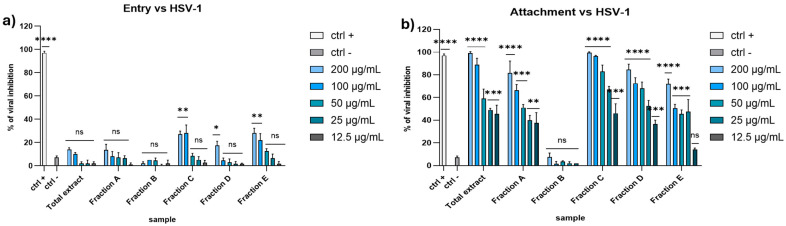
Entry (**a**) and attachment (**b**) assay against HSV-1. Concentrations are expressed in μg/mL. Positive control (ctrl +): heparin 1 mg/mL. Negative control (ctrl −): infected and untreated cells. **** *p* < 0.0001, *** *p* < 0.0009, ** *p* < 0.0061, * *p* < 0.0171, ns: not statistically significant.

As shown in Figure 4, it is clear that the extracts displayed a strong antiviral activity only in the attachment assay, suggesting their ability to interfere with the viral attachment on host cell membrane.

### 2.4. Molecular Networking Guided Dereplication of Fraction C from Diacronema lutheri

**Figure 5:**
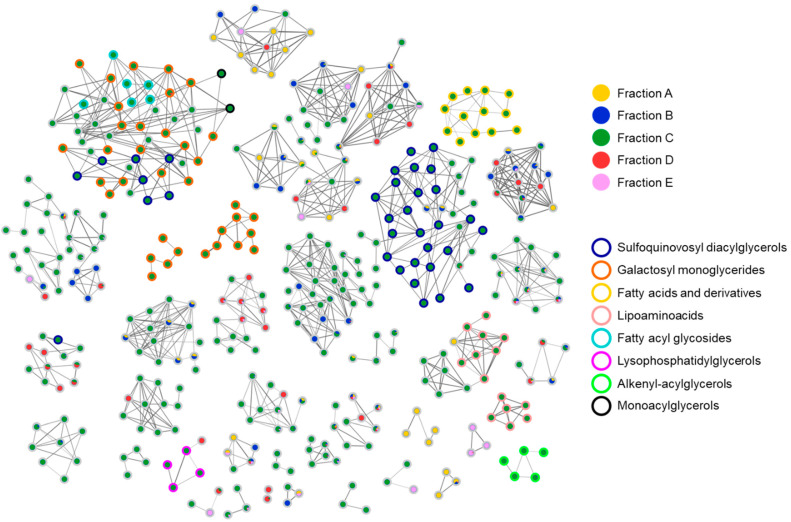
Molecular network of SPE fractions A–E from *Diacronema lutheri*. Nodes are visualized as pie charts showing metabolite distribution in SPE fractions. Border color of each node is mapped to the chemical class assigned by the integration of molecular networking and manual inspection of MS/MS fragmentation data.

To identify putative compounds responsible for the antiviral activity, liquid chromatography coupled with untargeted high-resolution tandem mass spectrometry (LC-HRMS^2^) were employed to chart the metabolite distribution in all SPE fractions and pinpoint molecular families unique (or almost unique) to the bioactive fraction C. LC-HRMS^2^ data were used to generate a molecular network (Figure 5) using the feature-based molecular networking workflow, which allows for the clustering of molecules based on MS/MS fragmentation similarity. Then, molecules are visualized in Cytoscape as nodes, connected through edges whose thickness is related to the MS^2^ spectra similarity. Combining manual inspection of MS^2^ data and dereplication against reference MS^2^ spectra from GNPS spectral libraries [28] enabled the identification of seven molecular families present almost exclusively in fraction C, namely sulfoquinovosyl acylglycerols, galactosyl monoglycerides, lipoaminoacids, fatty acyl glycosides, monoacylglycerols, lysophosphatidylglycerols, fatty acids and derivatives, and alkenyl-acylglycerols (Tables S1–S4).

**Figure 6:**
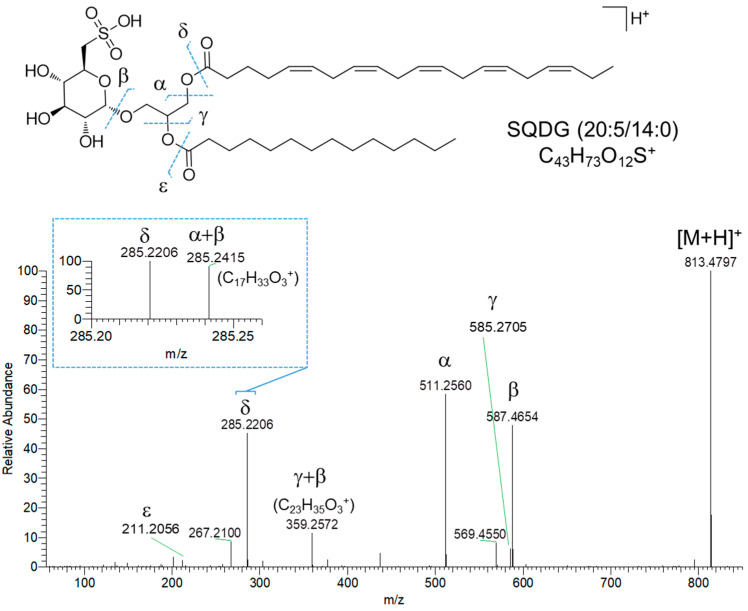
HR ESI-MS^2^ spectrum of the [M+H]^+^ pseudomolecular ion of SQDG (20:5/14:0) from *Diacronema lutheri*.

Sulfoquinovosyl di- and monoacylglycerols (SQDGs and SQMGs) represent the largest group of compounds in fraction C. SQMG and SQDG are a class of sulfolipids widespread in photosynthetic organisms, bearing a glycerol moiety anchoring one or two fatty acyl chains at the sn-1 and sn-2 positions and a 6′-sulfo-α-D-quinovosyl unit at the sn-3 position. The structures of 37 SQDGs and 7 SQMGs were predicted by HRMS^2^ analysis (Table 1). The product ion spectra of the [M+H]^+^ pseudomolecular ions of SQDGs and SQMGs display the fragment ion β, i.e., [M+H-226.0147]^+^, arising from the diagnostic neutral loss of the 6′-sulfo-α-D-quinovosyl residue (C_6_H_10_O_7_S) (Figure 6). Moreover, [M+H]^+^ ions of SQDG and SQMG undergo α and/or γ fragmentations due to carboxylic acid losses, thus leading to the identification of the fatty acyl substituents in all congeners (Figure 6). The regiochemical assignment of the fatty acyl chains on the sn-1 and sn-2 positions of the glycerol backbone in SQDG, was attempted based on the observation that fatty acid loss from the sn-1 position is favored over that from the sn-2 position, as previously reported [29]. The MS^2^ spectra of SQDGs and SQMGs feature additional product ions, such as (a) fragments generated after the fatty acid losses from the β fragment ion (Figure 6, α + β and γ + β fragmentations) and (b) acylium ions (Figure 6, δ and ε ions), thus providing useful information to further characterize the fatty acyl substituents.

**Figure 7:**
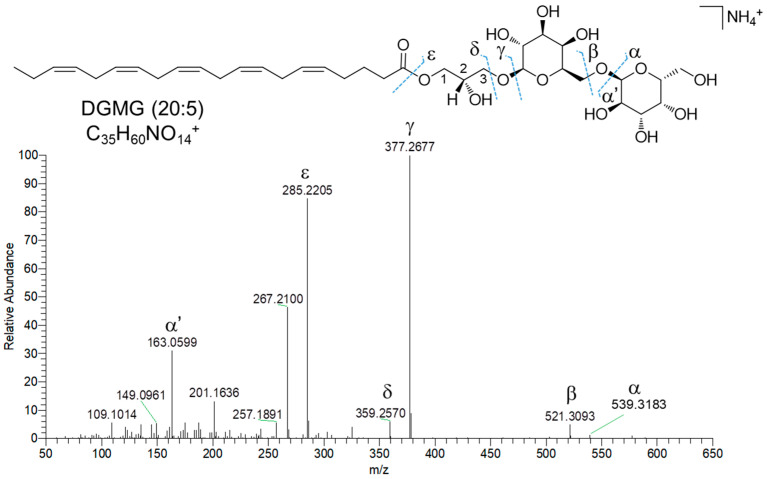
HR ESI-MS^2^ spectrum of the [M+NH_4_]^+^ ion of DGMG (20:5) from *Diacronema lutheri*.

Di- and monogalactosyl monoacylglycerols (DGMGs and MGMGs) are among the major constituents of fraction C, together with SQDGs and SQMGs. DGMGs and MGMGs are lyso-derivatives of di- and monogalactosyl diacylglycerols (DGDGs and MGDGs), where the glycerol backbone is typically esterified with a fatty acyl unit at the sn-1 position, although acyl migration may occur to give the more thermodynamically stable sn-2 isomer. In MGMGs, the galactosyl residue is bounded to the glycerol moiety through a β-glycosidic bond at the sn-3 position, whereas DGMGs display two α-1,6-glycosidic linked galactose units. Molecular networking based dereplication unveiled fraction C to contain 12 putative DGMGs and 13 putative MGMGs (Table 2). DGMGs and MGMGs were detected mainly as [M-H_2_O+H]^+^ and/or [M+NH_4_]^+^ adducts in full MS scans. MS^2^ spectra of DGMGs and MGMGs display fragment ions (Figure 7, β and δ fragmentations), arising from neutral losses of two and one hexose molecules (and the corresponding dehydrated forms—α and γ fragmentations, Figure 7), respectively, which were tentatively identified as galactose units. In addition, the presence of the hexose moiety was confirmed by the α’ fragment ion at *m*/*z* 163.0606 (C_6_H_11_O_5_)^+^ (Figure 7). The fragment ion γ corresponds to the monoacylglycerol unit, which, after glycerol loss, generates the ε acylium ion indicative of the fatty acyl chain in DGMGs and MGMGs (Figure 7).

**Figure 8:**
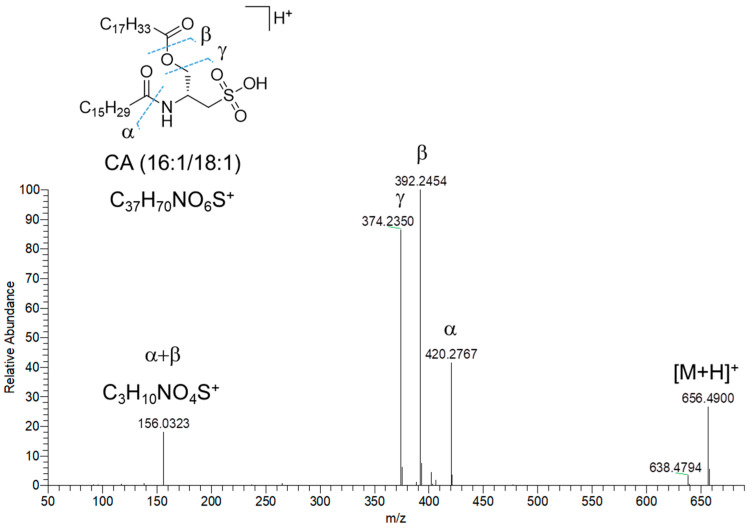
HR ESI-MS^2^ spectrum of the [M+H]^+^ pseudomolecular ion of cysteinolide L (CA(16:1/18:1)) from *Diacronema lutheri*.

Fifteen nodes in the molecular network of fraction C were annotated as amino lipids (AL), bearing a cysteinolic acid (CA) head group (Table 3). Only one AL was shown to have a leucine/isoleucine head group. CA-based amino lipids included N-acylated and N,O-acylated congeners. HR ESI-MS^2^ spectra of [M+H]^+^ ions of the N-acylated CA-based amino lipids feature (a) the diagnostic fragment ion corresponding to CA, i.e., *m*/*z* 156.0331 (C_3_H_10_NO_4_S^+^), arising from the neutral loss of the fatty acyl chain as ketene and (b) the acylium ion, generated after CA loss. Besides the presence of the CA fragment ion, the MS^2^ spectra of N,O-acylated CA-containing amino lipids, i.e., cysteinolides, are characterized by fragment ions resulting from (a) the loss of the acyl chain linked to the hydroxy group of CA, thus yielding the β and γ ions (Figure 8) and (b) the cleavage of the amide bond, which gives the α ion (Figure 8). The regiochemical assignment of the fatty acyl chains in cysteinolides was based on the favored fragmentation of the ester over the amide bond and the comparison with product ion spectra of cysteinolides described by Roman et al. [31]. Notably, MS-based structural elucidation of cysteinolides led to the identification of cysteinolides B and D, together with five novel variants, which were named K-O. So far, cysteinolides have not yet been described from microalgae, as they are being reported as bacterial metabolites [31].

## 3. Discussion

Haptophyta are unicellular flagellates that include about 500 species [32,33]. The genus *Diacronema* (also known as *Pavlova*) has shown to be of great interest in its use as feed in the aquaculture industry [33,34]. In this study, we present for the first time the antiviral activity of *D. lutheri*, showing a peculiar specific activity toward enveloped viruses. Fractionation of the total algal extracts and chemical analyses have then been performed to pinpoint the compound classes involved in the observed bioactivity. Thanks to the molecular networking guided dereplication, we identified the presence of seven molecular families almost exclusively present in fraction C, namely sulfoquinovosyl acylglycerols, galactosyl monoglycerides, lipoaminoacids, fatty acyl glycosides, monoacylglycerols, lysophosphatidylglycerols, fatty acids and derivatives, and alkenyl-acylglycerols. The glyceroglycolipids sulfoquinovosyl di- and monoacylglycerols (SQDGs and SQMGs) and di- and monogalactosyl monoacylglycerols (DGMGs and MGMGs) represent the largest groups of compounds in fraction C. These results are in accordance with the expected class of molecules in fraction C obtained from the SPE pre-fractionation methods of Cutignano et al. [35] in which glycolipids and phospholipids should be extracted by the elution solvent acetonitrile CH_3_CN/water H_2_O (70:30). Hulatt et al. in 2021 [19] also sequenced the genome of the *Diacronema lutheri NIVA*-4/92 and defined *D. lutheri* as a good model microalga for lipid biosynthesis studies [19].

The SQDGs, SQMGs, DGMGs and MGMGs have important biological properties such as immunomodulatory [36], anti-inflammatory [37], antioxidant [38,39] and anticancer [40,41] that are influenced by the saturation and length of their fatty acid chains [42,43]. Previous studies have shown the antiviral properties of glyceroglycolipids against HSV, as reported in Plouguerné et al. [44] and in Janwitayanuchit et al. [40]. In particular, DGMGs and MGMGs were capable of making the virus lose the ability to bind to the cells and inhibits their replication in vivo [45,46], while SQDGs were able to inhibit viral infection by combining with DNA to induce apoptosis in the infected cells [47].

Our findings demonstrate that *D. lutheri* has a strong antiviral potential in the early stage of infection, preventing the attachment and the entry of the virus into the host cell.

Currently, no precise mechanism has been elucidated, but we propose some potential modes of action based on the glyceroglylipids’ chemical structure. Due to their polarity, SQDGs and DGMGs could interact with and occupy the hydrophobic region of the HSV-1 glycoproteins like glycoprotein B (gB) and glycoprotein D (gD), which are essential for binding to the cell receptors. Moreover, SQDGs, for their amphipathic nature, could interact with envelope phospholipids and destabilize the integrity of the membrane needed for the infection. Marine microalgae represent a great source of natural products with a wide range of bioactivities, raising interest in the pharmacological and medical fields. Our study gives additional data on the lipid composition of the Haptophyta *Diacronema lutheri* and its possible application for specific viral infections.

Our results could be a starting point for exploiting marine microalgae in the production of antiviral compounds in order to have more of a chance in the design of new vaccines targeted at infections for which there is not yet an effective cure. Nowadays, despite the huge progress made, there are still many diseases that cannot be cured with vaccines, some of them include HIV infections, hepatitis C, hemorrhagic fever and herpes simplex [48]. The recent pandemic emergency caused by SARS-CoV-2, also known as COVID-19, highlighted the necessity to further invest in this field in order to discover antiviral extracts and bioactive natural products from the sea [5].

## 4. Materials and Methods

### 4.1. Cell Culturing and Harvesting

*Diacronema lutheri*, CCMP1325, was cultured in Guillard’s medium f/2 without silicates. Experimental culturing was performed in a 10 L polycarbonate bottle constantly bubbled with air filtered through 0.2 µm membrane filters. Initial cell concentrations were about 5000 cells/mL. At the end of stationary phase, the culture was centrifuged for 15 min at 4 °C at 3900× *g* and the pellets kept at −80 °C until chemical extraction.

### 4.2. Chemical Extraction and Fractionation

Microalgal cell pellets were extracted by sonication after 1 h of suspension in methanol 100% (1:5 *w*/*v*). After a centrifugation step at of 3400 rpm for 5 min at room temperature, the organic phase was dried at a reduced temperature with rotavapor (BÜCHI, Flawil, Switzerland). The extract was stored at −80 °C until fractionation.

Fractionation of 20 mg of extract was performed by SPE-solid phase extraction using CHROMABOND^®^ HR-X cartridges (6 mL/500 mg) (Macherey-Nagel) as in Cutignano et al. [30]. The cartridge was conditioned with 6 mL of methanol and equilibrated with 12 mL of distilled water. The extract was suspended in 1 mL of distilled water and sonicated for a few seconds before loading onto the column. The elution gradient was performed using different solvent conditions to obtain five fractions named A, B, C, D, E. The elution gradient was as follows, i. 18 mL of distilled water for the extraction of amino acid and saccharides (fraction A); ii. 24 mL of methanol CH_3_OH/water H_2_O (50:50) for the extraction of nucleosides (fraction B); iii. 18 mL acetonitrile CH_3_CN/water H_2_O (70:30) for the extraction of glycolipids and phospholipids (fraction C); iv. 18 mL 100% acetonitrile CH_3_CN for the extraction of free fatty acid and sterols (fraction D); v. 18 mL dichloromethane CH_2_Cl_2_/methanol CH_3_OH (90:10) for the extraction of triglycerides (fraction E).

### 4.3. Liquid Chromatography–High-Resolution Tandem Mass Spectrometry (LC-HRMS^2^)

The SPE fractions A–E were dissolved in CH_3_OH at a concentration of 1 mg/mL and analysed by LC-HRMS^2^ using a Thermo Scientific Q Exactive Focus Orbitrap mass spectrometer (Thermo Fisher Scientific Inc., Waltham, MA, USA) coupled to a Thermo Ultimate 3000 HPLC system equipped with an Hypersil C18 column (100 × 4.6 mm, 3 μm). The analytical column was maintained at 25 °C and eluted at a constant flow rate of 400 μL/min with H_2_O and CH_3_CN, both supplemented with 0.1% HCOOH, using the following gradient program: 10% CH_3_CN for 10 min (equilibration), 10% CH_3_CN for 1 min, 10% → 100% CH_3_CN over 30 min, 100% CH_3_CN for 10 min. MS spectra were acquired in the positive ion detection mode and HESI source parameters were set as follows: a sheath gas flow rate of 32 units N_2_, an auxiliary gas flow rate of 15 units N_2_, a spray voltage of 4.8 kV, a capillary temperature of 285 °C, an S-lens RF level of 55 and an auxiliary gas heater temperature of 150 °C. Full MS scans (300–2000  *m*/*z*) were recorded at a resolution of 70,000 and an AGC target of 1 × 10^6^. HRMS^2^ spectra were acquired in the data dependent acquisition mode at a resolution of 70,000 and an AGC target of 5 × 10^4^, setting three MS^2^ events after each full MS scan. HRMS^2^ scans were obtained with HCD fragmentation, using an isolation width of 2.0 *m*/*z*, a normalized collision energy of 15 units and an automated injection time.

### 4.4. Cytotoxicity

The effect of the extracts on cell viability was evaluated on Vero cells by 3-(4,5-dimethylthiazol-2-yl)-2,5-diphenyl-tetrazolium bromide (MTT) assay (Sigma-Aldrich, St. Louis, MO, USA). Raw extracts and fractions were first solved in dimethyl sulfoxide (DMSO) to obtain different concentrations to be tested for the bioactivity.

Vero cells (2 × 10^4^ cells/well) were seeded in 96-well plates and incubated O/N at 37 °C in a humidified atmosphere. The following day, cells were exposed to different concentrations of each extract for 24 h. After the treatment, MTT solution (0.5 mg/mL) was added to the Vero cells for 3 h. After 3 h, dimethyl sulfoxide (DMSO 0.5%) was added to each well to dissolve the formazan crystal. Cell viability was measured by recording the absorbance at the wavelength of 570 nm by TECAN M-200 reader (Tecan, Männedorf, Switzerland). The cell survival was calculated by the following formula:

(1) $$Cell\;viability\%=\frac{absorbance\;of\;treated\;samples\;}{absorbance\;of\;untreated\;samples\;}\times 100$$

### 4.5. Cell Lines, Viruses

African green monkey kidney epithelial cell lines (Vero CCL-81, Manassas, VA, USA; ATCC^®^) were cultivated in Dulbecco’s modified eagle medium (DMEM) supplemented with 10% heat-inactivated fetal bovine serum (FBS, Microgem, Naples, Italy), 2 mM L-glutamine (Microtech, Naples, Italy) and 100 IU/mL of penicillin-streptomycin solution (Himedia, Naples, Italy) and maintained at 37 °C in a humidified atmosphere with 5% CO_2_. Vero cells were used to propagate the following viruses: herpes simplex virus type 1 (HSV-1 strain SC16) and poliovirus type 1 (PV-1).

### 4.6. Antiviral Assay

The antiviral activity was evaluated through plaque reduction assay. Vero cells (1.3 × 10^5^ cells/well) were seeded in 24-well plates and incubated overnight. The monolayer was treated with different extract dilutions, ranging from 200 μg/mL to 3.1 μg/mL, and infected with viruses at 0.01 multiplicity of infection (MOI). Different schema of treatment was performed: (a) co-treatment assay, in which the monolayer was treated simultaneously with extract and virus and incubated for 1 h at 37 °C; (b) virus pre-treatment assay in which extract was let to act with virus at 0.1 MOI for 1 h at 37 °C. The solution was then diluted and used to infect the monolayer at 0.01 MOI for 1 h at 37 °C; (c) entry assay, in which the monolayer was treated with extract and virus (MOI 0.01) simultaneously, incubated for 1 h at 4 °C to allow the viral attachment but not the viral entry into the cells; (d) attachment assay, in which cells were first infected with virus (MOI 0.01), incubated at 4 °C for 1 h, then treated with extracts and incubated at 37 °C for 1 h to allow the entry phase.

At the end of each treatment, the cell monolayer was washed with citrate buffer (pH 3) to remove the non-penetrated virus and incubated for 24–48 h in DMEM supplemented with 3% carboxymethylcellulose (CMC). After the incubation, cells were fixed with 4% formaldehyde and stained with 0.5% crystal violet.

The percentage of viral inhibition was calculated by the formula:

(2) $$\%viral\;inhibition=\left(1-\frac{number\;of\;plaques\;in\;treated\;cells\;}{number\;of\;plaques\;in\;negative\;control\;}\right)\times 100$$

### 4.7. Feature-Based Molecular Networking (FBMN)

LC-HRMS^2^ raw data from SPE fractions A to E were first processed by MZMINE2 [49], setting the parameters reported in Table S5, as previously described [49]. In brief, following chromatogram building and deconvolution, peaks from the SPE fractions and blank sample (i.e., methanol used for sample dissolution) were aligned and the background spectrum was subtracted. Moreover, [M+Na–H]+, [M+K–H]+, [M+Mg−2H]2+, [M+NH3]+, [M-Na+NH4]+, [M+1, 13C]+ adducts were filtered out by setting the maximum relative height at 100%. Peaks without an associated MS/MS spectrum were finally filtered out from the peak list and processed data were then exported to the .mgf file for the FBMN workflow [50,51].

For FBMN analysis, the precursor ion mass tolerance was set to 0.02 Da and the MS^2^ fragment ion tolerance to 0.05 Da. A molecular network was then generated where edges were established to have a cosine score above 0.7 and more than 4 matched peaks. The analogue search mode was used by searching against MS^2^ spectra with a maximum difference of 100.0 in the precursor ion value. All matches kept between network spectra and library spectra were required to have a score above 0.7 and at least 6 matched peaks. The molecular network was visualized using Cytoscape software v. 3.8.2 [52]. The molecular network can be publicly accessed at https://gnps.ucsd.edu/ProteoSAFe/status.jsp?task=f76dce507a7f43b7ab2449edde9f04d6 (accessed on 8 April 2024).

### 4.8. Statistical Analysis

Statistical analyses were performed using GraphPad Prism (version 8.1.2, GraphPad Software Inc., San Diego, CA, USA). Arithmetic means ± the standard deviations (SD) were calculated and analyzed for statistical significance using a two-ways analysis of variance (ANOVA) following by Dunnett’s multiple comparison test. Differences at *p* < 0.05 were regarded as significant.

## Figures and Tables

**Table 1:** Sulfoquinovosyl di- and monoacylglycerols (SQDGs and SQMGs) identified in fraction C from *Diacronema lutheri*. Compounds are referred to by the LIPID MAPS abbreviations [30], where the first fatty acid in parenthesis is linked at the sn-1 position of the glycerol moiety, whereas the second fatty acid esterifies the sn-2 position.

	Compound	[M+H]^+^	*m*/*z*	*R*_t_ (min)	*Δ* (ppm)
Sulfoquinovosyl monoacylglycerols	SQMG (18:4/0:0)	C_27_H_45_O_11_S	577.2668	18.3	−1.6
	SQMG (18:4/0:0)	C_27_H_45_O_11_S	577.2668	18.5	−1.6
	SQMG (14:0/0:0)	C_23_H_45_O_11_S	529.2670	18.7	−1.4
	SQMG (14:0/0:0)	C_23_H_45_O_11_S	529.2670	19.0	−1.4
	SQMG (20:5/0:0)	C_29_H_47_O_11_S	603.2823	19.3	−1.7
	SQMG (16:1/0:0)	C_25_H_47_O_11_S	555.2825	19.6	−1.5
	SQMG (16:0/0:0)	C_25_H_49_O_11_S	557.2978	21.0	−2.3
Sulfoquinovosyl diacylglycerols	SQDG (8:3;O/14:0)	C_31_H_53_O_13_S	665.3191	19.2	−1.6
	SQDG (6:2;O/14:0)	C_29_H_51_O_13_S	639.3044	19.9	−1.3
	SQDG (9:3;O/14:0)	C_32_H_55_O_13_S	679.3345	20.9	−1.8
	SQDG (10:2;O/14:0)	C_33_H_59_O_13_S	695.3659	21.6	−1.7
	SQDG (12:4;O/14:0)	C_35_H_59_O_13_S	719.3655	22.2	−2.1
	SQDG (20:6;O/18:4)	C_47_H_71_O_13_S	875.4602	24.8	−0.8
	SQDG (20:5/18:5;O)	C_47_H_71_O_13_S	875.4602	24.8	−0.8
	SQDG (20:6/18:4)	C_47_H_71_O_12_S	859.4643	25.1	−2.0
	SQDG (20:5/18:5)	C_47_H_71_O_12_S	859.4643	25.1	−2.0
	SQDG (20:6/14:0)	C_43_H_71_O_12_S	811.4651	25.4	−1.2
	SQDG (16:2/14:0)	C_39_H_71_O_12_S	763.4651	25.7	−1.3
	SQDG (20:6;O/14:0)	C_43_H_71_O_13_S	827.4597	25.7	−1.5
	SQDG (16:2;O/14:0)	C_39_H_71_O_13_S	779.4598	26.0	−1.5
	SQDG (20:5/16:3)	C_45_H_71_O_12_S	835.4651	27.2	−1.2
	SQDG (20:5/18:4)	C_47_H_73_O_12_S	861.4807	27.7	−1.2
	SQDG (16:2;O/18:1)	C_43_H_77_O_13_S	833.5071	27.7	−1.0
	SQDG (20:3;O/14:0)	C_43_H_77_O_13_S	833.5071	27.7	−1.0
	SQDG (18:4/14:0)	C_41_H_71_O_12_S	787.4652	28.3	−1.1
	SQDG (20:5/14:0)	C_43_H_73_O_12_S	813.4806	28.6	−1.4
	SQDG (20:5/14:0)	C_43_H_73_O_12_S	813.4805	28.8	−1.5
	SQDG (18:4/16:1)	C_43_H_73_O_12_S	813.4805	28.9	−1.5
	SQDG (18:3/14:0)	C_41_H_73_O_12_S	789.4810	29.4	−0.9
	SQDG (22:6/14:0)	C_45_H_75_O_12_S	839.4964	29.4	−1.2
	SQDG (20:5/16:1)	C_45_H_75_O_12_S	839.4964	29.4	−1.2
	SQDG (14:0/14:0)	C_37_H_71_O_12_S	739.4654	29.6	−1.6
	SQDG (16:1/14:0)	C_39_H_73_O_12_S	765.4806	29.8	−1.5
	SQDG (14:1/16:0)	C_39_H_73_O_12_S	765.4806	29.8	−1.5
	SQDG (16:1/16:1)	C_41_H_75_O_12_S	791.4967	29.9	−1.0
	SQDG (18:2/14:0)	C_41_H_75_O_12_S	791.4967	29.9	−1.0
	SQDG (20:5/16:0)	C_45_H_77_O_12_S	841.5123	30.3	−0.8
	SQDG (20:3/14:0)	C_43_H_77_O_12_S	817.5124	30.3	−0.8
	SQDG (16:1/18:2)	C_43_H_77_O_12_S	817.5124	30.3	−0.7
	SQDG (15:0/14:0)	C_38_H_73_O_12_S	753.4810	30.4	−0.9
	SQDG (16:1/15:0)	C_40_H_75_O_12_S	779.4965	30.5	−1.0
	SQDG (17:1/14:0)	C_40_H_75_O_12_S	779.4965	30.5	−1.0
	SQDG (16:0/14:0)	C_39_H_75_O_12_S	767.4961	31.3	−1.6
	SQDG (18:1/16:0)	C_43_H_81_O_12_S	821.5430	32.3	−1.6

**Table 2:** Mono- and digalactosyl monoacylglycerols (MGMGs and DGMGs) identified in fraction C from *Diacronema lutheri*. Compounds are referred to by the LIPID MAPS abbreviations. ^a^ [M+H-H_2_O]^+^, ^b^ [M+NH_4_]^+^.

	Compound	[M+H]^+^	*m*/*z*	*R*_t_ (min)	*Δ* (ppm)
Monogalactosyl monoacylglycerols	MGMG (18:4;O)	C_27_H_43_O_9_ ^a^	511.2885	19.3	−3.2
	MGMG (20:5;O_2_)	C_29_H_45_O_10_ ^a^	553.2996	20.8	−2.0
	MGMG (20:5;O)	C_29_H_45_O_9_ ^a^	537.3049	20.0	−1.7
	MGMG (18:4)	C_27_H_48_O_9_N ^b^	530.3317	24.4	−1.2
	MGMG (20:5)	C_29_H_50_O_9_N ^b^	556.3471	25.4	−1.7
	MGMG (20:5)	C_29_H_50_O_9_N ^b^	556.3470	25.9	−1.9
	MGMG (18:3)	C_27_H_50_O_9_N ^b^	532.3470	26.0	−2.0
	MGMG (14:0)	C_23_H_48_O_9_N ^b^	482.3315	26.3	−1.8
	MGMG (16:1)	C_25_H_50_O_9_N ^b^	508.3472	27.1	−1.5
	MGMG (22:6)	C_31_H_49_O_9_	565.3351	27.5	−4.5
	MGMG (20:4)	C_29_H_52_O_9_N ^b^	558.3630	28.0	−1.1
	MGMG (18:2)	C_27_H_52_O_9_N ^b^	534.3630	28.3	−1.3
	MGMG (16:0)	C_25_H_52_O_9_N ^b^	510.3629	30.9	−1.5
Digalactosyl monoacylglycerols	DGMG (20:6)	C_35_H_55_O_14_	699.3576	18.5	−1.4
	DGMG (20:5;O)	C_35_H_55_O_14_ ^a^	699.3574	18.5	−1.7
	DGMG (20:5;O)	C_35_H_55_O_14_ ^a^	699.3574	18.9	−1.7
	DGMG (20:6;O)	C_35_H_53_O_14_ ^a^	697.3419	19.2	−1.5
	DGMG (20:5;O)	C_35_H_55_O_14_ ^a^	699.3574	19.8	−1.7
	DGMG (18:4)	C_33_H_58_O_14_N ^b^	692.3841	22.0	−1.6
	DGMG (20:5)	C_35_H_60_O_14_N ^b^	718.4002	22.8	−1.6
	DGMG (20:5)	C_35_H_60_O_14_N ^b^	718.3995	23.3	−1.8
	DGMG (14:0)	C_29_H_58_O_14_N ^b^	644.3838	23.2	−2.1
	DGMG (16:1)	C_31_H_60_O_14_N ^b^	670.3999	24.0	−1.3
	DGMG (18:3;O)	C_35_H_53_O_13_ ^a^	657.3472	24.0	−1.3
	DGMG (16:0)	C_31_H_62_O_14_N ^b^	672.4154	27.1	−1.6

**Table 3:** Amino lipids identified in fraction C from *Diacronema lutheri*. Compounds are referred to by the LIPID MAPS abbreviations. The first fatty acid in parenthesis is linked to the amino group of cysteinolic acid, whereas the second one esterifies the hydroxy function of cysteinolic acid. CA, cysteinolic acid.

	Compound	[M+H]^+^	*m*/*z*	*R*_t_ (min)	*Δ* (ppm)
*N*-acyl amino lipids	CA (18:4)	C_21_H_36_O_5_NS	414.2299	18.3	−2.4
	CA (14:0)	C_17_H_36_O_5_NS	366.2299	18.7	−2.8
	CA (16:1;O)	C_19_H_38_O_6_NS	408.2403	18.7	−2.8
	CA (20:5)	C_23_H_38_O_5_NS	440.2456	19.3	−2.0
	CA (16:1)	C_19_H_38_O_5_NS	392.2453	19.3	−3.0
	CA (16:0;O)	C_19_H_40_O_6_NS	410.2560	20.2	−2.6
	CA (22:6)	C_25_H_40_O_5_NS	466.2617	20.3	−2.3
	Leu/Ile (20:5)	C_26_H_42_O_3_N	416.3151	31.9	−2.0
Cysteinolides	CA (16:1;O/18:1) (cysteinolide K)	C_37_H_70_O_7_NS	672.4855	31.0	−1.9
	CA (16:1/18:1) (cysteinolide L)	C_37_H_70_O_6_NS	656.4911	31.4	−1.1
	CA (16:1;O/19:1) (cysteinolide M)	C_38_H_72_O_7_NS	686.5019	31.8	−0.8
	CA (17:1;O/18:1) (cysteinolide N)	C_38_H_72_O_7_NS	686.5019	31.8	−0.8
	CA (18:1;O/18:1) (cysteinolide B)	C_39_H_74_O_7_NS	700.5181	32.0	−1.0
	CA (16:0;O/18:1) (cysteinolide D)	C_37_H_72_O_7_NS	674.5016	32.1	−1.2
	CA (18:1/18:1) (cysteinolide O)	C_39_H_74_O_6_NS	684.5224	32.6	−1.1

## Data Availability

MS data are available in a publicly accessible repository at the following link: https://gnps.ucsd.edu/ProteoSAFe/status.jsp?task=f76dce507a7f43b7ab2449edde9f04d6 (accessed on 8 April 2024).

## Supplementary Materials

The following supporting information can be downloaded at: https://www.mdpi.com/article/10.3390/md23010012/s1, Table S1. Fatty acyl glycosides (FG) identified in fraction C from *Diacronema lutheri*; Table S2. Monoacylglycerols (MG) identified in fraction C from *Diacronema lutheri*; Table S3. Lysophosphatidylglycerols (LPG) identified in fraction C from *Diacronema lutheri*; Table S4. Alkenyl-acylglycerols (DG) identified in fraction C from *Diacronema lutheri*; Table S5. MZMINE2 parameters for MS raw data processing.
