# Supplementary material for: Antiviral Activity of the Marine Haptophyta Diacronema lutheri
Source: Mar Drugs. 2024 Dec 28;23(1):12. doi: 10.3390/md23010012 (PMC11766726; doi:10.3390/md23010012)
Supplement: Supplementary file 1 [file marinedrugs-23-00012-s001.zip › marinedrugs-3365745-supplementary.pdf]

Table S1. Fatty acyl glycosides (FG) identified in fraction C from *Diacronema lutheri*. Compounds are referred to by the LIPID MAPS abbreviations [1].

Fatty acyl glycosides				
**Compound**	**[M+H]+**	***m/z***	**R_t (min)**	**Δ (ppm)**
FG (16:2)	$C_{22}H_{39}O_7$	415.2679	18.9	-2.7
FG (16:2)	$C_{22}H_{39}O_7$	415.2679	19.6	-2.7
FG (9:1;O)	$C_{15}H_{27}O_8$	335.1691	19.7	-2.8
FG (16:2;O)	$C_{22}H_{39}O_8$	431.2631	19.7	-2.0
FG (14:0)	$C_{20}H_{39}O_7$	391.2680	24.5	-2.7
FG (16:1)	$C_{22}H_{41}O_7$	417.2835	25.5	-2.8

Table S2. Monoacylglycerols (MG) identified in fraction C from *Diacronema lutheri*. Compounds are referred to by the LIPID MAPS abbreviations [1]

Monoacylglycerols				
**Compound**	**[M+H]+**	***m/z***	**R_t (min)**	**Δ (ppm)**
MG (20:6)	$C_{23}H_{35}O_4$	375.2519	20.0	-2.9
MG (22:5)	$C_{25}H_{41}O_4$	405.2990	30.0	-2.3

Table S3. Lysophosphatidylglycerols (LPG) identified in fraction C from *Diacronema lutheri*. Compounds are referred to by the LIPID MAPS abbreviations [1]. [a] $[M+H-H_2O]^+$.

Lysophosphatidylglycerols				
**Compound**	**[M+H]+**	***m/z***	**R_t (min)**	**Δ (ppm)**
LPG (16:1)	$C_{22}H_{42}O_8P$ [a]	465.2605	21.7	-1.5
LPG (16:2)	$C_{22}H_{40}O_8P$ [a]	463.2447	20.4	-1.9
LPG (20:5)	$C_{26}H_{42}O_8P$ [a]	513.2607	20.1	-1.0

Table S4. Alkenyl-acylglycerols (DG) identified in fraction C from *Diacronema lutheri*. Compounds are referred to by the LIPID MAPS abbreviations [1].

Alkenyl-acylglycerols				
**Compound**	**[M+H]+**	***m/z***	**R_t (min)**	**Δ (ppm)**
DG (O-15:2/14:0) and/or DG (O-14:2/15:0)	$C_{32}H_{61}O_4$	509.4557	30.4	-1.4
DG (O-16:2/15:0) and/or DG (O-15:2/16:0)	$C_{34}H_{66}O_4$	537.4869	31.8	-1.6
DG (O-17:2/14:0) and/or DG (O-14:2/17:0)	$C_{34}H_{66}O_4$	537.4869	31.8	-1.6
DG (O-14:2/16:0) and/or DG (O-16:2/14:0)	$C_{33}H_{63}O_4$	523.4710	31.2	-2.0
DG (O-14:2/16:1) and/or DG (O-16:3/14:0)	$C_{33}H_{61}O_4$	521.4554	29.8	-2.1
DG (O-14:2/18:1) and/or DG (O-18:3/14:0)	$C_{35}H_{65}O_4$	549.4863	31.1	-2.7

Table S5. MZMINE2 parameters for MS raw data processing.

**Feature detection**	
MS level 1	1.0e5
MS level 2	1.0e2
ADAP chromatogram builder	□ group intensity threshold: 5.0e4<br>□ minimum highest intensity: 5.0e4<br>□ *m/z* tolerance: 0.005 *m/z* or 10 ppm
**Chromatogram deconvolution**	
baseline cut-off	□ minimum peak height: 9.0e4<br>□ peak duration range: 0.0 – 10.0 min<br>□ baseline level: 5.0e4<br>□ m/z range for MS2 scan pairing: 0.01 Da<br>□ RT range for MS2 scan pairing: 0.2 min
**Alignment**	
Join aligner	□ *m/z* tolerance: 0.002 *m/z* or 5 ppm<br>□ retention time tolerance: 0.1 min
**Identification**	
Adduct search<br>([M+Na–H], [M+K–H], [M+Mg−2H],<br>[$M+NH_3$], [$M-Na+NH_4$], [M+1, ^{13}C])	□ retention time tolerance: 0.1 min<br>□ *m/z* tolerance: 0.002 *m/z* or 5 ppm<br>□ max relative adduct peak height: 100%

References

1. Fahy E., Subramaniam S., Murphy R. C., Nishijima M., Raetz C. R., Shimizu T., et al. (2009). Update of the LIPID MAPS Comprehensive Classification System for Lipids1. *J. Lipid Res.* 50, S9–S14. doi: 10.1194/jlr.R800095-JLR200.
